# Follow-up of young adult monozygotic twins after simultaneous critical coronavirus disease 2019: a case report

**DOI:** 10.3389/fmed.2022.1008585

**Published:** 2022-09-29

**Authors:** Mateus V. de Castro, Monize V. R. Silva, Flávia B. Soares, Vivian R. Cória, Michel S. Naslavsky, Marilia O. Scliar, Erick C. Castelli, Jamile R. de Oliveira, Giuliana X. de Medeiros, Greyce L. Sasahara, Keity S. Santos, Edecio Cunha-Neto, Jorge Kalil, Mayana Zatz

**Affiliations:** ^1^Human Genome and Stem Cell Research Center, University of São Paulo, São Paulo, Brazil; ^2^Department of Genetics and Evolutionary Biology, Biosciences Institute, University of São Paulo, São Paulo, Brazil; ^3^Molecular Genetics and Bioinformatics Laboratory, Experimental Research Unit (Unipex), School of Medicine, São Paulo State University (UNESP), Botucatu, Brazil; ^4^Departamento de Clínica Médica, Disciplina de Alergia e Imunologia Clínica, Faculdade de Medicina da Universidade de São Paulo, São Paulo, Brazil; ^5^Laboratório de Imunologia, Instituto do Coração (InCor), LIM 19, Hospital das Clínicas da Faculdade de Medicina da Universidade de São Paulo, (HCFMUSP), São Paulo, Brazil; ^6^Instituto de Investigação em Imunologia—Instituto Nacional de Ciências e Tecnologia-iii-INCT, São Paulo, Brazil

**Keywords:** COVID-19, monozygotic twins, SARS-CoV-2, immunity, genetic variants

## Abstract

**Background:**

The influence of the host genome on coronavirus disease 2019 (COVID-19) susceptibility and severity is supported by reports on monozygotic (MZ) twins where both were infected simultaneously with similar disease outcomes, including several who died due to the SARS-CoV-2 infection within days apart. However, successive exposures to pathogens throughout life along with other environmental factors make the immune response unique for each individual, even among MZ twins.

**Case presentation and methods:**

Here we report a case of a young adult monozygotic twin pair, who caught attention since both presented simultaneously severe COVID-19 with the need for oxygen support despite age and good health conditions. One of the twins, who spent more time hospitalized, reported symptoms of long-COVID even 7 months after infection. Immune cell profile and specific responses to SARS-CoV-2 were evaluated as well as whole exome sequencing.

**Conclusion:**

Although the MZ twin brothers shared the same genetic mutations which may be associated with their increased risk of developing severe COVID-19, their clinical progression was different, reinforcing the role of both immune response and genetics in the COVID-19 presentation and course. Besides, post-COVID syndrome was observed in one of them, corroborating an association between the duration of hospitalization and the occurrence of long-COVID symptoms.

## Introduction

The ongoing global pandemic of coronavirus disease 2019 (COVID-19) caused by the SARS-CoV-2 virus has already affected the health of millions of people worldwide, with a significant number of deaths (1). Although older patients and those with comorbidities who are infected are more subject to unfavorable outcomes, reports of young people without chronic diseases who died from COVID-19 support the existence of genetic and immunological risk factors (2, 3). Also, several reports of identical twins deceased due to COVID-19 within days apart give further support to the influence of the host genome on COVID-19.

The first worldwide case of death from COVID-19, within 3 days apart, in one pair of adult unvaccinated MZ was reported in April 2020 in the United Kingdom. Aged 37, both twin sisters worked as nurses and had the same underlying health condition. Recently (2022), France’s famous twin Bogdanoff’s brothers died of COVID-19 6 days apart. Aged 72, the brothers had not been vaccinated against COVID-19 either.

The identification of genetic variants related to immune response, associated with higher susceptibility to the infection or severe COVID-19 has been the focus of numerous studies around the world (2, 4–8). Currently, genome-wide association studies (GWAS) have identified some genetic variants, including rare loss-of-function variants in genes involved in type I interferon (IFN) pathways (3, 9, 10) or missense variants that affect the activity of transmembrane serine protease 2 (3, 11, 12), that contribute to susceptibility or severe COVID-19, respectively. Here, we investigated a case of simultaneous critical COVID-19 in young adult MZ brothers in 2021, before being vaccinated. We present a comprehensive assessment of their innate and adaptive immunity, genetic profiling, and systemic biomarkers.

## Case presentation

**FIGURE 1:**
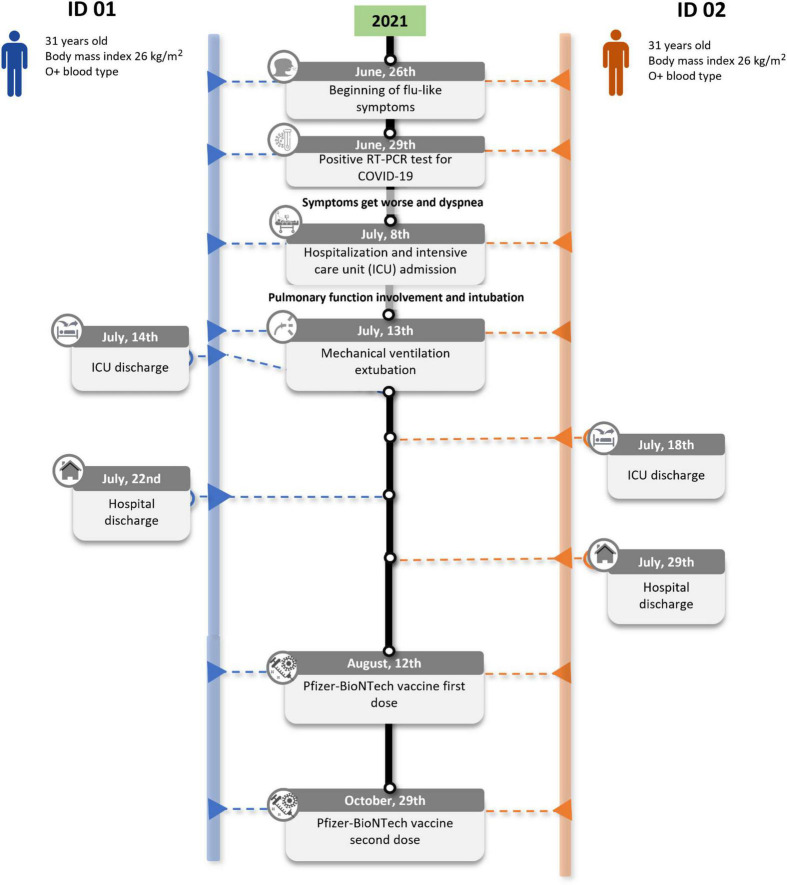
Clinical timeline of the major events of the twins’ case.

In June 2021, a 31-year-old Brazilian monozygotic twin brother pair (ID 01 and ID 02) started with cough and fever on the 26th. Both tested positive for SARS-CoV-2 infection on June 29th. During the following days symptoms worsened with dyspnea. From June 30th to July 4th, azithromycin was used. Blood oxygen saturation reached a critical level of 80% and the twin brothers were admitted directly to the intensive care unit on July 8th. They were intubated due to pulmonary involvement and were extubated after 5 days of mechanical ventilation (July 13th). The Gamma variant was the only SARS-CoV-2 variant identified in the region at the end of June 2021 (13) and it is known that this variant was associated with increased mortality risk and severity of COVID-19 cases in younger age groups, particularly in the unvaccinated population at the time (14). The twins received the same supportive ICU measures (sedation and proning). Also, at the hospital, both twins received the same treatment: dexamethasone (from July 9th to July 19th), to inhibit inflammation in the lungs. Due to detected resistant bacterial infections in both twins after extubation, they were treated with meropenem (from July 13th to July 20th). ID 01 was discharged on the 22nd of July and ID 02 7 days later. Both required respiratory physiotherapy for at least 3 months after hospital discharge. Seven months after the COVID-19 episode, ID 02 complained of persistent muscle fatigue, commonly associated with the post-COVID syndrome. The twins lived apart but worked at the same company as realtors. They did not have any known health conditions or comorbidities. The entire timeline of main events is presented in Figure 1.

Blood samples from the twins were collected in February 2022 (7 months after COVID-19 diagnosis and 4 months after getting a second dose of Pfizer-BioNTech COVID-19 vaccine) at our Research Center (HUG-CELL) for global immune profiling and genetic investigation. Peripheral blood mononuclear cells (PBMCs), plasma, and serum were obtained to perform the immunological assays and DNA for whole-exome sequencing (WES). Complementary clinical laboratory analyses were also performed in whole blood samples.

**FIGURE 2:**
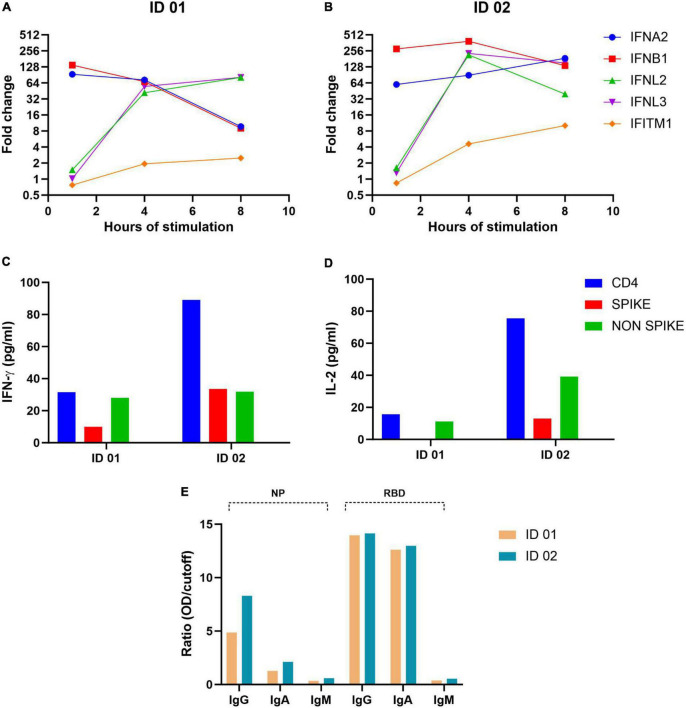
Global immune profiling of the twin volunteers, 7 months after the COVID-19 episode. **(A,B)** Type I/III IFN production by PBMCs after toll-like receptor (TLR) stimulus. **(C,D)** IFN-γ and IL-2 production by PBMC when stimulated by SARS-CoV-2 peptides. **(E)** Serological assays for SARS-CoV-2 IgA, IgG, and IgM through ELISA for the receptor-binding domain (RBD) and nucleocapsid protein (NP).

Surface immunophenotyping of PBMC was performed by flow cytometry (Table 1). The twins displayed normal frequencies of CD3+, CD4+, and CD8+ T-cells, monocytes, NK cells, and lymphocytes B as expected in healthy donors (15). The type I/III IFN production by PBMCs after toll-like receptor (TLR) stimulus (double-stranded RNA Poly I:C), was evaluated for 1, 4, and 8 h. Although there was heterogeneity in IFN or IFN-induced gene expression, the twins presented an early and strong (FC = 20 or higher) mRNA expression of at least two of the five types of I/III IFN analyzed. Thus, no failure in the innate IFN response was observed. The production of interferon-gamma (IFN-γ) and interleukin-2 (IL-2) by PBMC, after stimulation by SARS-CoV-2 peptides, was also evaluated. Similar results were observed in both twins, for CD4 + T lymphocyte responses. ELISA serological assays were performed for SARS-CoV-2 IgA, IgG, and IgM for the receptor-binding domain (RBD) and nucleocapsid protein (NP) to assess their humoral immune response. The antibody profiles of SARS-CoV-2 IgA, IgM, and IgG were virtually identical between the MZ twin brothers. The global immune profiling of the twins is presented in Figure 2.

**TABLE 1:** PBMCs immunophenotyping of the twin volunteers, 7 months after COVID-19 episode.

ID	CD3 + T-cells	CD4 + T-cells	CD8 + T-cells	Monocytes	NK cells	B-cells
01	56.2	63.5	31.1	7.7	8.9	9.3
02	57.5	57.7	35.3	8.0	8.8	7.7
Healthy*	45–70	25–60	5–30	10–30	5–10	5–15

*Reference parameters values for individuals in the same age range (15).

Hematologic and systemic parameters of the post-COVID phase (Table 2) revealed great similarity between the MZ twins, except for a very slight increase in creatine phosphokinase (an enzyme specific to muscle tissues, which may increase after muscle injuries) and ferritin (an acute phase reactant that can increase its serum concentration during inflammation), presented by ID 02. These findings might be related to the fatigue reported only by this twin. Likewise, both presented mild changes in erythrocyte sedimentation rate, a parameter that may be increased in different inflammatory conditions.

**TABLE 2:** Blood test parameters of the volunteers, 7 months after the COVID-19 episode.

Variables	ID 01	ID 02	Reference values
**Erythrogram**			
Erythrocyte/red blood cell (RBC) count (millions/mm^3^)	4.64	4.57	4.30–5.70
Hemoglobin concentration (g/dL)	14.8	14.1	13.5–17.5
Hematocrit (%)	42.0	42.0	39.0–50.0
Mean cell/corpuscular volume—MCV (μ^3^)	90.5	91.9	81.2–95.1
Mean cell hemoglobin—MCH (%)	31.9	30.9	26.0–34.0
Mean cell hemoglobin concentration—MCHC (pg)	35.2	33.6	31.0–36.0
RBC distribution width—RDW (%)	11.6	11.3	8.0–15.5
Erythrocyte sedimentation rate—ESR (mm/1 h)	**12**	**18**	0–6
**Leukogram**			
Leukocyte/white blood cell (WBC) count/mm^3^	4,310	4,840	3,500–10,500
Neutrophil count/mm^3^	2,030.01	2,574.88	1,700.00–8,400.00
Eosinophil count/mm^3^	349.11	208.12	50.00–420.00
Basophil count/mm^3^	60.34	48.40	0.00–105.00
Lymphocyte count/mm^3^	1,560.22	1,669.80	900.00–3,150.00
Monocyte count/mm^3^	310.32	338.80	140.00–1,260.00
**Coagulation parameters**			
Platelet count/mm^3^	311,000	304,000	140,000–400,000
Prothrombin time (s)	10.2	10.6	9.6–12.0
Activated partial thromboplastin time—APTT (s)	32.5	32.1	22.7–32.5
D dimer (μg/mL)	0.15	0.17	0–0.50
**Homeostasis parameters**			
C-reactive protein—CRP (mg/dL)	0.12	0.14	0–0.60
Ferritin (ng/mL)	331.0	**512.1**	25.0–400.0
Lactate dehydrogenase—LDH (U/L)	193	198	0–250
**Parameters of tissues’ functions**			
B-type natriuretic peptide–BNP (pg/mL)	<5	<5	<100
Troponin T (ng/mL)	<0.003	0.003	0.000–0.030 (negative)
Creatine phosphokinase—CPK (U/L)	186	**211**	0–190
Glutamic-oxaloacetic transaminase—GOT (U/L)	34	28	0–50
Glutamic pyruvic transaminase—GPT (U/L)	47	33	0–50
Urea (mg/dL)	27	28	10–50
Creatinine (mg/dL)	0.8	0.9	0.7–1.2

The parameters out of the reference values were highlighted in red.

WES was performed in peripheral blood DNA with the Illumina NovaSeq platform at HUG-CELL facilities. The identical twins do not carry any rare variants in genes associated with inborn errors (IE) of Toll-like receptor 3 (TLR3) and IFN regulatory factor 7 (IRF7) dependent type I IFN immunity, which underlies life-threatening COVID-19 pneumonia (5, 16). Also, we did not detect any copy number variation (CNV) in IE genes. The Neanderthal-derived genetic variant rs35044562 (17) was not detected in the twins. However, we detected two rare missense variants (with a mean CADD score > 20), one in the *BTK* gene (NM_000061:exon8:c.G684A:p.M228I) carried in homozygosity and one in the *NFKB2* gene (NM_002502:exon22:c.T2531C:p.V844A) carried in the heterozygous state, which may be linked to their increased risk of developing severe COVID-19. In addition, we analyzed the genotypes and haplotypes (Supplementary Table 1) of the HLA cluster in the MHC region by using a hla-mapper (version 4) (18) to optimize read alignment along the MHC region. Interestingly, the twins present the alleles HLA-A*02:01 and HLA-E*01:01 (both carried in the heterozygous state), which were associated with the high severity of COVID-19.

## Discussion

Twin studies are important to investigate the contribution of genetics vs. the environment, in the susceptibility or resistance to infectious diseases as well as their pathomechanisms. Moreover, the study of the monozygotic ones may represent a powerful approach to further explore the immunological factors that contributed to the host defense. Beyond the host genotype, the individual immune response plays a determining role in the success or failure against SARS-CoV-2 (15). The immune repertoire which is also somatically defined by mutations occurring at later stages of development could justify different disease courses, and/or outcomes even in monozygotic twins (19, 20).

The genetic causes responsible for the clinical variability associated with COVID-19 remain the subject of investigation. Worldwide genomic studies of large cohorts of individuals with different clinical manifestations have been published, suggesting the involvement of different genetic variants responsible for greater susceptibility or resistance to SARS-CoV-2. GWAS identified a potential effect of variants in the *SLC6A20*, *LZTFL1*, *CCR9*, *FYCO1*, *CXCR6*, and *XCR1* genes as responsible for greater susceptibility to SARS-CoV-2 (2, 19) in addition to variants in the genes *REXO2*, *C11orf71*, *NNMT*, and *CADM1*, involved in the immune response (21). Additionally, variants in many genes involved with the innate immune response seem to be involved in the susceptibility/predisposition to severe cases of COVID-19 such as those involved in the IFN and TLRs pathways, as well as the *ACE2* and *TMPRSS2* genes involved in virus entry into the cell (3, 7, 22). Variants in the genes *IL1B, IL1R1, IL1RN, IL6, IL17A, FCGR2A*, and *TNF* which encode cytokines would also have a possible relation with disease susceptibility and cytokine storm development. The Human Leukocyte Antigen (HLA) gene cluster and genes associated with the Major Histocompatibility Complex (MHC) are important candidates for the mechanisms of innate and adaptative immunity and susceptibility to COVID-19 infection and manifestation (23).

Interestingly the identified heterozygous *NFKB1* missense variant (NM_002502:exon22:c.T2531C:p.V844AI) and the hemizygous missense variant in the *BTK* gene (NM_000061:exon8:c.G684A:p.M228I) are the central hubs that connect proinflammatory signaling pathways for survival, proliferation, cytokine production, and lymphocyte development. Interestingly, variants in both genes have been reported in primary antibody immunodeficiencies (24, 25). However, since these variants were not studied at the protein or functional level, their pathogenicity is yet to be determined. Genetic variant in chromosome 3, previously associated with high severity cases of COVID-19 and inherited from Neanderthals (rs35044563), was not detected in both volunteers (17). Regarding the HLA complex, the twins present the alleles HLA-A*02:01 and HLA-E*01:01 (both carried in the heterozygous state), which were associated with high severity of COVID-19 (13).

The global immune profiling assays, after 7 months of the COVID-19 episode, revealed great similarities between the MZ twins. It is known that the failure to elicit a strong type I IFN response contributes to severe COVID-19 (14). Infections trigger massive T cell expansion, leading to the skewing of the TCR repertoire due to antigen−specific T cell expansion. A low clonotype sharing between MZ twins with rheumatoid arthritis that were mismatched for SARS-CoV-2 infection suggests an immune repertoire reshaping might be induced after COVID-19 (26). However, clonality and alterations of T-cell receptors’ repertoires were partly associated with immune activation mediated by IFN type I and III (27) and here both twins displayed early and strong I/III IFN responses. The production of cytokines IFN-γ and IL-2 by T lymphocytes, when stimulated by SARS-CoV-2 peptides, was expressive. IL-2 and IFN-γ, which play a critical role in the activation of macrophages and other immune cells related to viral clearance, were found to be specific biomarkers of SARS-CoV-2 cellular response (28). The virus-specific antibody responses showed a vigorous IgA and IgG similar response in both twins. Since these analyses were done post-vaccination, it is not clear whether it was the viral infection or the vaccines that stimulated the production of these antibodies but it is clear there is no deficient humoral response. Taken together, regarding the immune response, all parameters analyzed were practically identical among the MZ twins.

Although both twins required intensive care and mechanical ventilation for 5 days, ID 02 required longer hospitalization and presented long-term symptoms consistent with long COVID. After 7 months of follow-up, twin ID 02 reported persistent muscle weakness and fatigue, while twin ID 01 referred to a return to his usual state of health. Muscle dysfunction (intense fatigue) is among the most reported symptoms of the post-COVID syndrome (17). The laboratory values obtained at 7 months demonstrated that twin ID 02 had an elevation in ferritin and CPK but otherwise had similar hematological and functional parameters relative to twin ID 01. Importantly, the twins were not on any medications or supplements. The CK levels at admission are reported to be higher in those subjects who later experience more severe outcomes and were associated with a worse prognosis (20) while severe to critical COVID-19 patients showed higher ferritin levels compared to mild to moderate COVID-19 patients (29). Thus, the slightly abnormal ferritin and CPK from ID 02 even after 7 months post-hospitalization might play a role in the pathogenesis of post-acute sequelae of COVID-19.

## Conclusion

This case study on two young-adult monozygotic twins simultaneously infected with SARS-CoV-2, both requiring ICU care but with different periods of clinical progression suggests the contribution of both immune response and the genetics in the COVID-19 presentation and course. Besides, the post-COVID syndrome was observed in one of them, corroborating an association between the duration of hospitalization and the occurrence of long-COVID symptoms.

## Data availability statement

The original contributions presented in this study are included in the article/Supplementary material, further inquiries can be directed to the corresponding author.

## Ethics statement

The studies involving human participants were reviewed and approved by CAAE 34786620.2.0000.5464. The patients/participants provided their written informed consent to participate in this study. Written informed consent was obtained from the individual(s) for the publication of any potentially identifiable images or data included in this article.

## Author contributions

MC and MVRS: data curation, investigation, formal analysis, visualization, and writing—original draft. FS: data curation and investigation. VC and EC-N: writing—review and editing. MN: conceptualization, formal analysis, investigation, methodology, software, and writing—review and editing. MOS and EC: formal analysis, investigation, methodology, software, and writing—review and editing. JO and GS: methodology and writing—review and editing. KS: investigation, visualization, and writing—review and editing. JK: funding acquisition, resources, and writing—review and editing. MZ: conceptualization, funding acquisition, project administration, writing—original draft, and writing—review and editing. All authors contributed to the article and approved the submitted version.
