# Corrigendum: Follow-up of young adult monozygotic twins after simultaneous critical coronavirus disease 2019: a case report

**DOI:** 10.3389/fmed.2023.1185833

**Published:** 2023-04-28

**Authors:** Mateus V. de Castro, Monize V. R. Silva, Flávia B. Soares, Vivian R. Cória, Michel S. Naslavsky, Marilia O. Scliar, Erick C. Castelli, Jamile R. de Oliveira, Giuliana X. de Medeiros, Greyce L. Sasahara, Keity S. Santos, Edecio Cunha-Neto, Jorge Kalil, Mayana Zatz

**Affiliations:** ^1^Human Genome and Stem Cell Research Center, University of São Paulo, São Paulo, Brazil; ^2^Department of Genetics and Evolutionary Biology, Biosciences Institute, University of São Paulo, São Paulo, Brazil; ^3^Molecular Genetics and Bioinformatics Laboratory, Experimental Research Unit (Unipex), School of Medicine, São Paulo State University (UNESP), Botucatu, Brazil; ^4^Departamento de Clínica Médica, Disciplina de Alergia e Imunologia Clínica, Faculdade de Medicina da Universidade de São Paulo, São Paulo, Brazil; ^5^Laboratório de Imunologia, Instituto do Coração (InCor), LIM 19, Hospital das Clínicas da Faculdade de Medicina da Universidade de São Paulo, (HCFMUSP), São Paulo, Brazil; ^6^Instituto de Investigação em Imunologia—Instituto Nacional de Ciências e Tecnologia-iii-INCT, São Paulo, Brazil

**Keywords:** COVID-19, monozygotic twins, SARS-CoV-2, immunity, genetic variants

In the published article, there was an error in the author list, and author Giuliana X. de Medeiros was erroneously excluded. The corrected author list appears below.

Mateus V. de Castro^1†^, Monize V. R. Silva^1†^, Flávia B. Soares^1, 2†^, Vivian R. Cória^1^, Michel S. Naslavsky^1, 2^, Marilia O. Scliar^1, 2^, Erick C. Castelli^3^, Jamile R. de Oliveira^4, 5^, Giuliana X. de Medeiros^4, 5^, Greyce L. Sasahara^5^, Keity S. Santos^4, 5, 6^, Edecio Cunha-Neto^4, 5, 6^, Jorge Kalil^4, 5, 6^ and Mayana Zatz^1, 2*^

The authors apologize for this error and state that this does not change the scientific conclusions of the article in any way. The original article has been updated.

