# Supplementary material for: Follow-up of young adult monozygotic twins after simultaneous critical coronavirus disease 2019: a case report
Source: Front Med (Lausanne). 2022 Sep 29;9:1008585. doi: 10.3389/fmed.2022.1008585 (PMC9558815; doi:10.3389/fmed.2022.1008585)
Supplement: Supplementary file 1 [file Table_1.pdf]

Supplementary Material

Supplementary Table 1. HLA class I and II alleles of the volunteers.

Gene	Allele 1	Allele 2
HLA-A	*68:02:01	*02:01:01
HLA-B	*42:01:01	*44:02:01
HLA-C	*17:01:01	*05:01
HLA-E	*01:01:01	*01:03:02
HLA-G	*01:01:01	*01:01:02
MICA	*004:01	*008:01
MICB	*018:01:01	*019:01:01
HLA-DOB	*01:01:01	*01:01:01
